# Investigating intimate partner violence as a dementia risk factor through co‐creation of research design and knowledge translation deliverables with survivors and sector partners

**DOI:** 10.1002/alz.093580

**Published:** 2025-01-09

**Authors:** Cheryl L Wellington

**Affiliations:** ^1^ University of British Columbia, Vancouver, BC Canada

## Abstract

**Background:**

Approximately 30% of women worldwide experience intimate partner violence (IPV). Although as many as 92% report impacts to the head and/or strangulation that raise clinical suspicion of brain injury (BI), there are no evidence‐based methods to document IPV‐BI in this vulnerable population, no clinical practice guideline, and insufficient understanding about long‐term risks including Alzheimer’s Disease and Related Dementias (ADRD). Although traumatic brain injury (TBI) is an established ADRD risk factor, little is known about attributable risk of ADRD due to IPV in either military or civilian populations. Our study aims to improve diagnosis of BI in survivors of IPV in a manner sensitive to the needs of this vulnerable population, and to raise awareness of the importance of considering IPV‐BI as a potential ADRD risk factor.

**Methods:**

We were recently approved by the USA Department of Defence to launch a prospective observational study that leverages collaborative research at three clinical sites in British Columbia Canada that reach ethnically and geographically diverse participants. We will assess acute and chronic plasma biomarkers as diagnostic tools and will also use an integrated knowledge translation (iKT) framework including community participation to deliver a living clinical practice guideline for IPV‐BI and trauma‐informed guidance for ADRD researchers to specifically ask about IPV in clinical history.

**Results:**

Thus far, we have worked with multiple stakeholders to co‐create a novel Case Report Form suitable for use in real‐life clinics to collect IPV history including head impact and strangulation events, and have pilot data on several plasma biomarkers relevant to ADRD that, for some cases, suggest IPV may lead to accelerated brain aging and biomarker patterns consistent with AD.

**Conclusions:**

We will discuss how extensive feedback from persons with lived experience and stakeholder groups was essential to design a feasible community‐based study, as common data elements now almost routine in ADRD and TBI studies needed to be reworked to consider the unique needs of IPV survivors in participating in research.

